# Accuracy of Tempo Judgments in Disk Jockeys Compared to Musicians and Untrained Individuals

**DOI:** 10.3389/fpsyg.2021.709979

**Published:** 2021-10-05

**Authors:** Nicholas E. V. Foster, Lauriane Beffa, Alexandre Lehmann

**Affiliations:** ^1^Department of Otolaryngology Head and Neck Surgery, McGill University, Montreal, QC, Canada; ^2^International Laboratory for Brain, Music and Sound Research (BRAMS), Montreal, QC, Canada; ^3^Center for Research on Brain, Language and Music (CRBLM), Montreal, QC, Canada

**Keywords:** DJ, training, practice, auditory, tempo, rhythm, beat, absolute perception

## Abstract

Professional disk jockeys (DJs) are an under-studied population whose performance involves creating new musical experiences by combining existing musical materials with a high level of temporal precision. In contemporary electronic dance music, these materials have a stable tempo and are composed with the expectation for further transformation during performance by a DJ for the audience of dancers. Thus, a fundamental aspect of DJ performance is synchronizing the tempo and phase of multiple pieces of music, so that over seconds or even minutes, they may be layered and transitioned without disrupting the rhythmic pulse. This has been accomplished traditionally by manipulating the speed of individual music pieces “by ear,” without additional technological synchronization aids. However, the cumulative effect of this repeated practice on auditory tempo perception has not yet been evaluated. Well-known phenomena of experience-dependent plasticity in other populations, such as musicians, prompts the question of whether such effects exist in DJs in their domain of expertise. This pilot study examined auditory judgments of tempo in 10 professional DJs with experience mixing by ear, compared to 7 percussionists, 12 melodic instrumental musicians, and 11 untrained controls. Participants heard metronome sequences between 80 and 160 beats per minute (BPM) and estimated the tempo. In their most-trained tempo range, 120–139 BPM, DJs were more accurate (lower absolute percent error) than untrained participants. Within the DJ group, 120–139 BPM exhibited greater accuracy than slower tempos of 80–99 or 100–119 BPM. DJs did not differ in accuracy compared to percussionists or melodic musicians on any BPM range. Percussionists were more accurate than controls for 100–119 and 120–139 BPM. The results affirm the experience-dependent skill of professional DJs in temporal perception, with comparable performance to conventionally trained percussionists and instrumental musicians. Additionally, the pattern of results suggests a tempo-specific aspect to this training effect that may be more pronounced in DJs than percussionists and musicians. As one of the first demonstrations of enhanced auditory perception in this unorthodox music expert population, this work opens the way to testing whether DJs also have enhanced rhythmic production abilities, and investigating the neural substrates of this skill compared to conventional musicians.

## Introduction

An essential element of musical rhythm is the “beat” or regular rhythmic pulse to which we often dance or clap. While listening to music, the beat—a periodic, isochronous pulse—is readily perceived from the music even in musically untrained individuals, and even when the rhythmic patterns present in the music do not always coincide with the pulse (Cannon and Patel, 2021). Together, the beat and the rate of the beat (the musical tempo, commonly expressed in beats per minute, “BPM”) represent fundamental temporal features in music perception (Geiser et al., 2014). In turn, tempo plays an important role in musical expression and appreciation (Palmer, 1997; Quinn and Watt, 2006). Although a melody’s identity can be defined in terms of relative structure, such as pitch and time intervals, the absolute “surface features” of tempo, key, and timbre are important attributes by which we can identify and remember particular musical performances (Levitin and Cook, 1996; Schellenberg et al., 2014; Jakubowski et al., 2018) and musical works (Clynes and Walker, 1986; Bailes and Barwick, 2011).

Precise perception and synchronization with the beat are important for performing musicians, as well as for other expert populations whose performance involves manipulating and combining music, such as disk jockeys (DJs). However, although musicians have been highly studied as a model of experience-dependent plasticity in various aspects of auditory perception, DJs have only recently gained attention as an expert population in this domain (Butler and Trainor, 2015). Consequently, much remains to be discovered about how the particular experience of DJs may lead to similar or unique patterns of ability compared to conventionally trained musicians.

Musical training is associated with greater accuracy of temporal perception and sensorimotor synchronization. Compared to untrained individuals, musicians have improved detection of timing differences for individual sound durations as well as for temporal manipulations embedded within sound sequences (Yee et al., 1994; Rammsayer and Altenmüller, 2006). Musicians are also sensitive to smaller changes of tempo than nonmusicians (Ellis, 1991; Sheldon, 1994). Percussionists, whose training emphasizes rhythmic timing, exhibit particular advantages over non-percussionists in performing fine temporal discrimination (Ehrlé and Samson, 2005; Manning and Schutz, 2016) as well as in sensorimotor timing synchronization and reproduction tasks (Krause et al., 2010; Cicchini et al., 2012; Cameron and Grahn, 2014).

Given the importance of musical rhythm and timing in DJ performance, and a comparable degree of long-term practice between DJs and many musicians (e.g., Butler and Trainor, 2015), we may predict that DJs also possess experience-dependent expertise on rhythmic tasks. A central skill in DJ performance is combining and transitioning between different musical works in a rhythmically seamless way. In its most long-standing form, this involves modulating the playback of a sound recording by directly interacting with a vinyl record and a variable-speed turntable in order to bring two songs into precise temporal alignment at a common tempo, and further adjusting playback as necessary to maintain synchrony (Broughton and Brewster, 2003, p. 50). This approach therefore relies on a combination of precise temporal perception to detect asynchrony and sensorimotor control to apply appropriate corrections to the record or turntable. In the context of an entire DJ performance, this process is aided by memory for the musical structure of each particular song, including its rhythm and beat tempo (Brewster and Broughton, 2000, p. 8). Similar to musicians, for whom tempo is often indicated explicitly on musical scores and controlled during practice by the use of a metronome, DJs are aided in choosing and synchronizing music by knowledge of the explicit tempo value of each song (Broughton and Brewster, 2003, p. 83). Whereas enhanced rhythmic perception and sensorimotor precision have been measured in DJs, as reviewed below, the accuracy of tempo judgments in DJs remains unexamined and is the focus of the present study.

Current evidence for training-related expertise in rhythmic perception in DJs comes from research by Butler and Trainor (2015). In that study, professional club DJs, percussionists, and untrained controls were tested on their ability to detect deviations in auditory rhythmic patterns. Compared to the untrained group, both the DJ and percussionist groups showed enhanced detection of deviations from “on beat” timing for a probe stimulus following an isochronous pattern and a brief silent period. The DJ group’s detection sensitivity was comparable to the percussion group across all levels of deviation. The authors interpret the enhanced performance on this task to reflect both greater sensitivity to small timing discrepancies and more accurate memory for rhythm through the brief silent period.

Although memory for musical tempo has not yet been studied in DJs, several studies have demonstrated that absolute tempo is remembered for highly familiar songs in untrained individuals, and this tempo memory also exists for newly heard songs when tested at shorter intervals (e.g., minutes or hours). An early demonstration of this phenomenon was made by Levitin and Cook (1996), who found that when asked to sing, hum, or whistle from memory a popular song they knew well, most participants’ renditions had a tempo within 8% of the recorded version. Subsequent studies have found similar accuracy in the range of 3–18% for highly familiar music in participants unselected for musical experience (Jakubowski et al., 2015, 2016, 2018). Additional support for the stability of absolute tempo representation comes from the observation that songs sung by mothers to their infants deviate in tempo by only 3% across sessions (Bergeson and Trehub, 2002), and evidence for memory of absolute tempo for unfamiliar birdsong (Roeske et al., 2020) and periodic environmental sounds like footsteps (Boltz, 2010) show that this ability generalizes beyond music. Evidence for a link between absolute tempo ability and musical training currently remains unclear and limited by methodological approach. A study of musicians and nonmusicians by Gratton et al. (2016) showed that untrained individuals can learn a novel absolute tempo scale but did not show clearly higher performance in musicians compared to untrained controls. In that study, the spacing between unique items was quite large (12%) and may not have had sufficient resolution to find differences between the training groups. Another study that attempted to relate individual differences in musical training to tempo accuracy of remembered familiar music did not find a significant relation (Jakubowski et al., 2018), but the number of participants was small. Nonetheless, these studies validate the idea that at least at a coarse level, tempo is remembered regardless of musical experience.

While a basic fidelity of musical tempo memory has been established in previous research, DJs present an ideal population to test whether this ability can be enhanced by sustained practice. In turn, this approach offers an opportunity to broaden the empirical description of an under-studied model of experience-dependent plasticity in professional DJs. Hence, in consideration of the importance of musical tempo to DJ performance, the goal of the present pilot study was to characterize tempo perception in highly trained DJs and compare its accuracy with conventionally trained experts in rhythm and music, i.e., percussionists and melodic musicians. The inclusion of three expert groups, with similar training duration but different training characteristics, allowed us to assess the specificity of tempo estimation ability to different training profiles. Tempo perception was tested by presenting a range of metronome tempos and measuring the accuracy of tempo estimates in groups of DJs, percussionists, melodic musicians, and untrained individuals. The results were examined to determine the effects of training and tempo upon the accuracy of this judgment. We predicted that DJs would show an enhanced ability to discern the tempo of metronome sounds compared to untrained individuals, and similar accuracy when compared to percussionists and melodic musicians.

## Materials and Methods

### Participants

Forty participants (23 male, mean age 29.3years, SD 8.4) participated in the study, comprising 10 professional DJs with experience mixing by ear, 7 percussionists, 12 melodic instrumental musicians, and 11 untrained controls. Table 1 summarizes the characteristics of the study groups. DJs were required to have at least 10years of experience DJing for at least 6h per week and to mix music without the aid of synchronizing software (e.g., using turntables). Melodic musicians and percussionists were required to have at least 10years of musical practice for at least 6h per week. Individuals in the untrained group were required to have less than 3years of musical training or DJ experience. Participants completed a questionnaire to verify their history of musical or DJ experience. Group matching was not achieved for gender or age. The DJ group consisted of only male participants, whereas the other groups had between 27 and 72% male participants. Mean age was greater in the DJ group and did not differ among the other groups [*F*(3,36)=13.9, *p*<0.001; Tukey-corrected pairwise comparisons with DJ group *p*≤0.014]. These potential group confounds were addressed in additional analyses as described later. The experimental protocol was approved by the Comité d’éthique de la recherche en arts et en sciences at the University of Montreal. Participants gave informed consent and were compensated for their time.

**Table 1:** Participant characteristics.

				Age^*^		Years DJ experience		Years music experience^†^	
Group	*n*	n Male	% Male	Range	Mean (SD)	Range	Mean (SD)	Range	Mean (SD)
DJ	10	10	100.0	30–52	39.2(7.7)	9–40	19.6(11.1)	0–12	3.3(5.1)
Percussionist	7	5	71.4	22–45	29.9(7.6)	0–5.5	0.8(2.1)	11–27	16.9(5.1)
Musician	12	5	41.7	20–33	26.2(4.8)	0–0.5	0.1(0.2)	10–28.5	16.7(5.2)
Untrained	11	3	27.3	20–28	23.7(3.3)	0–0	0.0(0.0)	0–3	1.0(1.1)

* *Mean age is greater in the DJ group and does not differ among the other groups [F(3,36)=13.9, p<0.001; Tukey-corrected pairwise comparisons with DJ group p ≤ 0.014]*.

† *Music experience is based on years of formal practice on a musical instrument*.

### Stimuli

Stimuli in the tempo estimation task consisted of isochronous patterns of a metronome sound. The full set of 81 stimulus rates included every integer BPM rate from 80 to 160 (i.e., inter-onset intervals of 750ms at 80 BPM to 375ms at 160 BPM). The metronome sound was 25ms in duration, with a strong initial transient, and having spectral energy concentrated between 500Hz and 3kHz. Each stimulus was created from the metronome sound repeated at the BPM rate with silence in the intervening samples. The total duration of each stimulus varied between 5 and 10s and was chosen randomly on each trial.

### Procedure

At the beginning of the testing session, participants completed a questionnaire to assess their history of DJ and musical experience. DJs were also asked to indicate the range of musical tempos they played most often.

The tempo estimation task then took place in a soundproof audio booth. The task was implemented using the Psychophysics Toolbox extensions (Psychtoolbox; RRID:SCR_002881) in Matlab (RRID:SCR_001622), running on a MacBook Pro computer connected to an RME Fireface audio interface, and Behringer HA8000 headphone amplifier. Participants heard audio stimuli *via* Beyerdynamic DT 770 PRO 250 Ohm headphones and received visual prompts and feedback on a computer display.

Before testing commenced, the tempo estimation task was explained to the participant. Music recordings were played to illustrate specific tempo values, covering the range used in the task: “Halo” by Beyonce (80 BPM), “Stayin’ Alive” by the Bee Gees (104 BPM), “Raise Your Glass” by Pink (122 BPM), “Viva la Vida” by Coldplay (138 BPM), and “Happy” by Pharrell Williams (160 BPM). These song tempos were determined by automatic analyzer (Traktor software, Native Instruments, Berlin, Germany) and confirmed *via* online databases^1^^,^^2^ and manually by an experienced DJ.

In the tempo estimation task, each trial started with a 500Hz warning beep for 1s, followed by 500ms of silence, followed by the metronome stimulus. Upon completion of the metronome stimulus, the participant was prompted to estimate the stimulus tempo in BPM using the computer keyboard. After the participant’s response, the actual stimulus tempo was displayed on the screen for 1s. The participant was then presented with a random number between 20 and 100 and asked to count backward from that number, out loud, until a subsequent stop message 5s later. In combination, the feedback and subsequent counting phase were intended to help orient the participants (especially the untrained group) to the absolute tempo scale, while interfering with relative comparison of successive trial stimuli by increasing irrelevant load on auditory working memory. Finally, a 3s delay followed each trial.

Participants completed four practice trials at randomly selected tempos and then had the opportunity to ask questions before the testing phase began. In the testing phase, participants completed 36 trials of the tempo estimation task at tempos randomly selected from the full set of 81 tempos between 80 and 160 BPM, with no tempos repeated. To reduce fatigue, at an interval of every nine trials, the participant was allowed to take a pause before resuming the task. The entire task was about 30–45min in duration, including instructions and practice trials.

### Analyses

Given the stimulus tempo range of 80–160 BPM, tempo responses of <20 BPM were considered to be at risk of typing errors and were discarded prior to analysis (N=8 trials out of 1,440 total). The remaining responses had a range of 63–172 BPM.

These BPM responses were then converted to absolute percent error, i.e., 100^*^abs[(response BPM—actual BPM)/(actual BPM)]. When stimulus BPM was used as an independent variable in analysis, it was binned into four tempo ranges: 80–99 BPM, 100–119 BPM, 120–139 BPM, and 140–160 BPM.

Statistical analysis was performed using R (R Core Team, 2019, RRID:SCR_001905). In consideration of the relatively small sample size and lack of balance between groups (McNeish and Harring, 2017), a mixed-effects model was used to test the effects of group and tempo range on absolute percent error scores on the BPM judgment task, using lmerTest (Kuznetsova et al., 2017; RRID:SCR_015656) with lme4 (Bates et al., 2015; RRID:SCR_015654). Group and tempo range were parameterized using sum-to-zero contrasts, and a random effect of participant was added to account for the hierarchical structure of the data. Omnibus tests of the main effects and their interaction were performed on this model *via* the car package (Fox and Weisberg, 2011), yielding F and *p* values calculated from Wald tests using Kenward-Roger approximated degrees of freedom (Halekoh and Højsgaard, 2014) and type 3 sum of squares. Following a significant interaction effect, the emmeans R package (Lenth, 2020; RRID:SCR_018734) was used to test post-hoc comparisons between groups within a tempo range (24 pairwise comparisons) and differences between tempo ranges within a group (24 pairwise comparisons). The post-hoc tests were corrected for 48 multiple comparisons using the Sidak method (Abdi, 2007).

In order to assess potential confounds between group, age, and gender, additional mixed-effects models were run in specific groups to test age and gender as predictors of absolute percent error scores. The first model included age, tempo range, and their interaction as predictors, with participant as a random effect. The second model included gender, tempo range, and their interaction, with participant as a random effect. Both of these models were tested in the untrained group alone, as well as with a pooled sample composed of the untrained, percussion, and musician groups.

## Results

**Figure 1:**
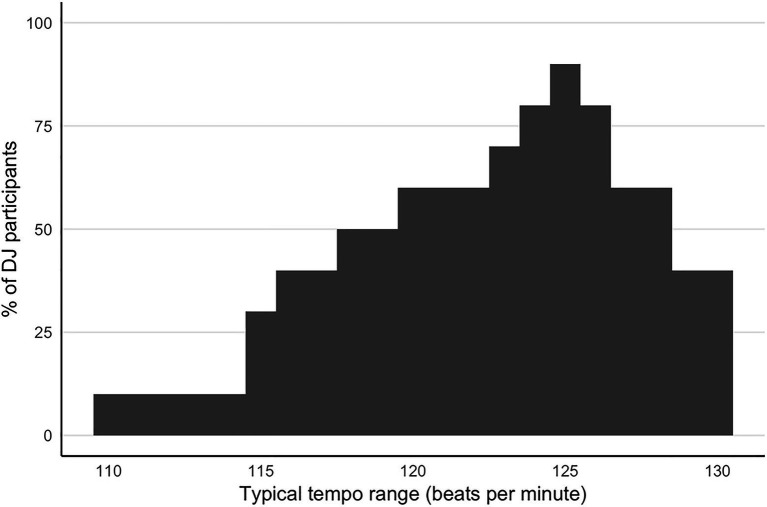
Distribution of tempos reported as typically played by participants in the DJ group. Each participant reported a range of tempos (minimum and maximum), and the distribution shows at each BPM what proportion of DJ participants included this tempo in their range.

Participants in the DJ group were asked to report the typical range tempos of the music they play. The distribution of those ranges is shown in Figure 1. A total of 72.9% of this distribution falls into the range 120–130 BPM, with a peak at 125 BPM, and the remaining 27.1% of the distribution falls into the range 110–119 BPM.

**Figure 2:**
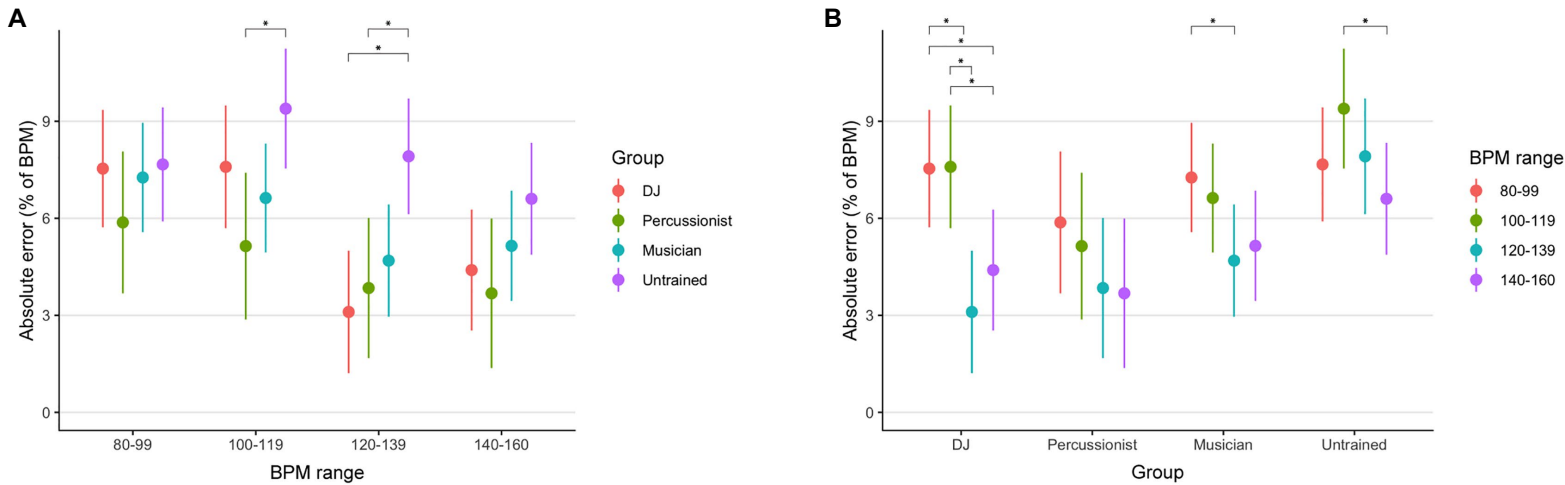
Mean accuracy of tempo judgments by participant group and tempo range. In panel **(A)**, the values are organized by group, and Sidak-corrected significance of pairwise differences between tempo ranges within groups is indicated. In panel **(B)**, values are organized by tempo range and Sidak-corrected significance of pairwise differences between groups is indicated. Error bars indicate 95% confidence intervals of the estimated marginal means.

The mixed-effects analysis of absolute percent error on BPM judgments found main effects of group [*F*(3, 36.02)=5.67, *p*=0.003] and tempo range [*F*(3, 1389.56)=19.86, *p*<0.001], and interaction between group and tempo range [*F*(9, 1389.55) = 2.70, *p*<0.001]. Mean error by group and tempo range is shown in Figure 2. Post-hoc comparisons showed that in their most-trained tempo range of 120–139 BPM, DJs were more accurate than untrained participants (DJ error 3.10%, untrained error 7.91%, *p* <0.001). Additionally, within the DJ group, this 120–139 BPM range exhibited greater accuracy than for slower tempos of 80–99 (error 7.54%, *p*<0.001) or 100–119 BPM (error 7.59%, *p*<0.001). When compared with the groups having conventional musical training, DJs did not differ in accuracy compared to percussionists or melodic instrumental musicians on any BPM range (*p*≥0.84).

Similar to DJs, percussionists were more accurate than untrained individuals on both the 100–119 (percussionist error 5.14%, untrained error 9.39%, *p*=0.017) and 120–139 BPM ranges (percussionist error 3.84%, untrained error 7.91%, *p*=0.018). Melodic musicians did not differ in accuracy with any other group, although within 120–139 BPM, the difference in accuracy between musicians and controls was near significance (120–139 BPM musicians vs. controls, *p*=0.065; other comparisons *p*≥0.255).

Because of the greater mean age and exclusively male composition of the DJ group, it was important to test whether age or gender could have imparted a confounding effect on performance differences found in the DJ group. For both age and gender, additional analyses on error scores were performed in the untrained group alone, as well as across a pooled sample of the untrained, percussion, and musician groups. No significant effects of age or gender were found. In untrained individuals, there was no main effect of gender [*F*(19.07)=0.19, *p*=0.671] nor an interaction between gender and tempo range [*F*(3,379.56)=1.4750, *p*=0.221]. Similarly, across all the non-DJ groups, there was no main effect of gender [*F*(128.03)=2.45, *p*=0.13] nor an interaction between gender and tempo range [*F*(3,1044.16)=0.59, *p*=0.62]. In the untrained group, in which ages ranged from 20 to 28years, there was no main effect of age [*F*(19.03)=0.12, *p*=0.73] nor an interaction between age and tempo range [*F*(3,378.75)=0.67, *p*=0.57]. Across all the non-DJ groups, in which ages ranged from 20 to 45years, there was a marginal effect of age [*F*(128.31)=4.10, *p*=0.052] and no interaction between age and tempo range [*F*(3,1046.09)=0.18, *p*=0.912]. Here, even given the possibility of a main effect of age, there is no evidence for a specificity of effect to any particular tempo range, in contrast to the performance differences noted for the DJ and percussion groups earlier.

## Discussion

These pilot results provide evidence that DJs and percussionists are more accurate at estimating auditory metronome tempo than untrained individuals. In DJs, this increased accuracy showed a specificity to the musical tempo ranges that participants reported as typical for their practice (120 to 139 BPM) and approached the previously reported perceptual discrimination threshold for tempo comparisons (Drake and Botte, 1993). The results add to the evidence that DJs are a highly trained expert population who exhibit heightened musical perception, comparable to percussionists, even though the nature of their craft and training is different from conventionally trained musicians (Butler and Trainor, 2015). As both the tempo estimation task and the study population are relatively novel, in the following discussion, we will examine several aspects of the task with consideration to both the baseline of tempo perception in the general untrained population and the tempo-specific enhancement observed in DJs.

The task in the present study asked participants to estimate the tempo of metronome stimuli using numeric values of beats per minute. To orient participants for whom this scale is unfamiliar, several pieces of popular music were played before the experiment, which together covered the range of tested tempos. Additionally, the true tempo was displayed following each trial, after the participant recorded their response. These elements of the procedure made it feasible to test tempo estimation accuracy using a continuous, concrete response scale in both untrained and trained individuals. An interfering step was added after the feedback to guard against performing the task simply using auditory working memory. This was done because the display of the true tempo of each trial could also make it feasible to perform the task in sequential and relative manner, simply comparing the tempo of the present item with the label and perceptual memory of the previous item; this relative aspect of absolute perceptual tasks exists even in the absence of feedback (Lockhead, 2004). To interfere with this effect, participants were required to count backward, out loud, for a 5s period between each trial. Nonetheless, part of the increased accuracy in tempo estimation by the trained groups that we observe could reflect a greater resilience of memory from trial to trial and decreased interference from the backward counting.

The perceptual and associative operations involved in the task may be useful to consider separately (Kim and Knösche, 2017), especially because much of the existing research on absolute tempo memory does not involve explicit labeling of tempo values. The tempo judgment task in our study asks participants to respond to each stimulus by estimating its tempo in beats per minute and hence involves a combination of perceptual extraction of the absolute tempo and an associative process of labeling this tempo with a numeric value. We may imagine that the cumulative experience of practicing as a DJ or musician improves both the perceptual and associative abilities involved in the absolute tempo memory phenomenon examined here, but potentially to different extents or with different training trajectories.

Some studies of absolute tempo perception have used tasks without any explicit tempo labeling, and their results may help elucidate the perceptual component. Halpern and Müllensiefen (2008) exposed participants having various degrees of musical experience to unfamiliar melodic excerpts, then in a subsequent testing phase, presented tempo-modified and original versions of these melodies along with novel melodies. The tempo-modified versions, which had been speeded or slowed by 15–20%, were rated as less familiar than the original versions, and this effect was not modulated by musical experience. A more recent study by Schellenberg et al. (2014) has replicated this result using a greater tempo change. Furthermore, the basic ability to remember the tempo of musical pieces appears to emerge quite early in development, as shown in infants using tempo alterations of 25% (Trainor et al., 2004).

In quantitative terms, the untrained participants in the present study had a mean absolute error of about 7.5% over the full range of tempos; DJs had the best error rate in the 120–139 BPM range, at 3%. Although no previous studies have tested absolute tempo labeling on an explicit BPM scale, the magnitude of error is comparable to that found in other types of tasks of absolute tempo memory. Studies that involve reproducing a familiar tune’s tempo, whether by singing, humming, or tapping, generally report error values between 3 and 18% in participants unselected for musical experience. For example, Levitin and Cook (1996) found that 72% of the productions on two consecutive trials came within 8% of the actual tempo. Two studies of tapping to involuntary musical imagery, or “earworms,” found mean differences of 11% (Jakubowski et al., 2015) and 8% (Jakubowski et al., 2018) as compared to the song’s canonical sound recording. When tunes are generally well known but not specifically selected by each participant, Jakubowski et al. (2016) found somewhat higher error levels of 18% when tapping to the imagined song, but 8% when listening and adjusting the playback rate of the actual song recording. The lowest value of 3% was found for songs sung by mothers to infants, measured between different renditions by the same mother (Bergeson and Trehub, 2002).

One common aspect of the latter literature is that participants are evaluated on tempo memory for auditory material that is highly familiar, either as popular music canon (e.g., Levitin and Cook, 1996; Jakubowski et al., 2016) or personal familiarity (e.g., Bergeson and Trehub, 2002; Jakubowski et al., 2015). As mentioned previously, another experimental approach familiarizes participants with novel musical material (Halpern and Müllensiefen, 2008; Schellenberg et al., 2014; Schellenberg and Habashi, 2015) or non-musical periodic environmental sounds (Boltz, 2010). These studies tested whether tempo manipulations reduce subsequent familiarity ratings, with the inference that lower ratings for tempo-modified items provide evidence of absolute tempo memory for the original stimulus. The tempo shifts in these tasks were 15–60% and therefore help establish an upper bound on absolute tempo resolution for unfamiliar auditory material. This may be particularly relevant for the untrained participants in the present study, because while the examples of several specific popular song tempos may have been familiar to them, both the absolute BPM tempo scale and the specific metronome stimuli were novel to them prior to the present experiment.

A study of absolute tempo labeling and production by Gratton et al. (2016) has several important points of comparison with the present results. In that study, seven metronome stimuli at systematically spaced tempos between 71 and 143 BPM were familiarized in two groups of musicians and nonmusicians, and then tested with tasks of absolute identification and finger tapping production. The steps between the stimulus levels were about 12% by tempo, and in the perceptual identification task, nonmusicians selected the correct tempo item 45% of the time, whereas musicians had an identification accuracy rate of 53% that was not significantly different from nonmusicians. Those results validate the idea that untrained individuals can learn an unfamiliar absolute tempo labeling scale, as they were similarly asked to do in the present study. The lack of a clear musical training effect in the study by Gratton and colleagues likely reflects the wide granularity of their tempo intervals, which were 9–16 BPM apart, whereas the spacing of 1 BPM in the present study provides a finer resolution to estimate absolute tempo perception accuracy. An additional important aspect of the Gratton study is that their task involved explicit labeling of the test stimuli on an ordinal tempo scale. The participants were able to learn this novel tempo scale and then label items on the scale, which requires both the perceptual component of extracting the tempo on an absolute scale and the associative component of selecting a discrete name for the value (Kim and Knösche, 2017), as in the present task.

Another notable finding in the absolute tempo perception study by Gratton and colleagues is a U-shaped distribution of accuracy. In studies of absolute perception, this is often referred to as the “bow” or edge effect, where identification is more accurate toward the minimum and maximum of the tested range (Brown et al., 2008). The present results appear to be consistent with this effect in the untrained group, where the intermediate 100–119 BPM range has greater error than the fastest 140–160 BPM range (Figure 2B), whereas the pattern is not seen for the trained groups. Indeed, participants having DJ or musical training may have prior, stable BPM referents in the intermediate tempo ranges that would counteract this bow effect.

Finally, the error level achieved by DJs in the 120–139 BPM range approaches the just noticeable difference (JND) of 2–3% previously reported for tempo discrimination tasks using isochronous auditory sequences (Drake and Botte, 1993). This performance is notable both for its high accuracy and its tempo specificity. The 120–139 BPM tempo range coincides with the tempos reported by the same participants as most frequently played during their DJ experience (Figure 1) and is also in concordance BPM values previously reported as typical for dance music played by DJs (Moelants, 2008). Several models of absolute perception emphasize the importance of stable memory for specific referents on the absolute scale. In the ANCHOR model by Petrov and Anderson (2005), these referent anchors serve the mapping from the perceptual continuum to the response scale, are competitively selected among when a response is needed, and provide a stable basis to estimate the actual response value. Subsequent research by others has reinforced the importance of longer-term absolute referents over short-term relative comparisons (SAMBA model; Brown et al., 2008, 2009) and determined that bow effect (decreased performance in the middle of the scale range compared to the extremes) is diminished when intermediate values are frequently encountered (Kent and Lamberts, 2016). In DJs, high familiarity with the tempos of music in their repertoire, especially those songs that are most frequently played, likely provides stable anchors referents to support highly accurate tempo estimation in this task. Additionally, as DJs are often aware of the numeric BPM value of their most familiar music, their experience could serve to provide both stable anchors that refine an internal continuum of perceptual tempo, and also the mapping to BPM values when an explicit value is to be estimated.

### Limitations

This is a pilot study with 40 participants, and consequently, there is likely a diminished sensitivity to detect group differences and a greater influence of individual participants on the analytic results. A mixed-effects model was used in order to minimize bias in model estimates and obtain statistical power with a relatively small sample with unbalanced groups (McNeish and Harring, 2017). For a more detailed profile of absolute tempo accuracy in DJs and percussionists, a larger study will be necessary.

As this is a cross-sectional study, it cannot be excluded that a preexisting disposition to precise tempo estimation influenced people to become DJs or percussionists, rather than (or in addition to) the training of these individuals honing their tempo perception abilities. There is suggestive evidence that even a single week of DJ training can improve rhythm perception (Butler and Trainor, 2015). However, a longer longitudinal study is needed to confirm if tempo or rhythm perception accuracy is driven primarily by practice effects rather than predisposition in DJs.

While this study demonstrates the phenomenon of increased absolute tempo ability in DJs, it is not able to dissociate several presumed components of this ability. The accuracy of tempo memory that is measured presumably depends in turn upon the perceptual resolution for tempo, the extent and quality of one’s internal representation of tempo scale, and associations between anchors on this scale and explicit tempo value labels. The degree to which greater accuracy observed in DJs and percussionists may result from enhancement of one of these abilities (e.g., knowledge of labels for scale anchors) or a coordination of multiple abilities is unknown. To tease apart these abilities and better define the key mechanisms involved, future research on this phenomenon would benefit from comparing these groups with untrained individuals on additional tasks that do not involve explicit tempo labeling. At a low level, a tempo JND task (e.g., Drake and Botte, 1993) could test whether greater sensitivity to fine tempo differences accounts for some variation in tempo memory fidelity. A more intermediate task, like reproduction of the tempo of familiar songs (as in Jakubowski et al., 2016), could better define the stability of anchors on the absolute tempo scale, without the requirement that these anchors be associated with explicit tempo values.

### Directions for Future Research

With very little existing empirical research on DJs, many interesting questions remain about the skill profile and underlying experience-dependent plasticity in this expert population. In addition to dissociating the components of absolute tempo estimation and confirming their refinement by DJ training, as described in the previous section, future research may examine the potential role of different technical approaches in DJing and seek a more detailed profile of tempo and rhythm abilities in DJs.

Through much of the history of DJing, proficiency has involved the development of sufficient perceptual and motor skills to synchronize different musical recordings, “by hand” and “by ear” (Brewster and Broughton, 2000). As we are interested in the perceptual expertise of DJs, the present study required DJ participants to have experience with playing vinyl records and mixing them by ear. In recent years, technological aids are more readily available to DJs, and many of these are aimed at easing the task of synchronizing different pieces of music. At its most basic, this support includes tempo counters in mixer hardware and extends to automated synchronization between songs provided by DJ hardware or software (Broughton and Brewster, 2003, p. 97). The extent to which tempo judgment accuracy in our DJ group may generalize to other DJs whose training embraces this synchronization assistance is unknown. It may be that tempo labeling ability remains comparably accurate due to the experience of consistent software-provided tempo displays while practicing and performing, whereas detection of beat asynchrony may be less accurate for DJs who rely upon automated synchronization of the music they play.

Given the importance of rhythm to music in general, and to the musical practice of DJs, there is a constellation of related rhythmic perception and production skills that may also be improved in DJs. The previous study by Butler and Trainor (2015) has already demonstrated that temporal changes in rhythmic sequences are better detected in highly trained DJs (at a comparable sensitivity to percussionists) and are improved in untrained individuals who undergo 1 week of DJ practice. Detecting fine temporal structure is important for DJs when synchronizing music, as the tempo must be matched and the phase kept in alignment. One beat-based perceptual task that would be well suited to evaluating DJs’ expertise is found in the Beat Alignment Test battery of Iversen and Patel (2008), which assesses sensitivity to detect a mismatch of tempo or phase in a metronome overlaid on music. Butler and Trainor (2015) also highlight the relevance of DJs’ sensorimotor expertise, finding that finger tapping to the beat of their auditory stimuli aided the perceptual detection of rhythmic deviations. Indeed, neural systems involved in motor control are believed to contribute to the precision of rhythm perception when there is a regular beat-based structure (Grube et al., 2010; Teki et al., 2011). Fine temporal motor control is also essential for DJs when they manually manipulate music playback to bring two songs into temporal alignment and apply corrections to maintain synchrony. Tasks measuring temporal sensorimotor production, such as those found in the BAASTA (Bégel et al., 2018) and H-BAT (Fujii and Schlaug, 2013) rhythmic assessment batteries, offer a means for future research to test whether these abilities are enhanced in DJs.

## Conclusion

In sum, the present work highlights that DJs are a highly trained population having expertise in musical tempo perception that is comparable to percussionists, but with a unique performance profile shaped by their most frequently practiced musical repertoire. Parallels between DJs and musicians, both in their extensive experience and enhanced perception, point to many potential avenues for future research in this new model of experience-dependent plasticity, with the opportunity to more broadly understand the effects and neural bases of sustained practice in the brain.

## Data Availability Statement

The raw data supporting the conclusions of this article will be made available by the authors, without undue reservation.

## Ethics Statement

The studies involving human participants were reviewed and approved by the Comité d’éthique de la recherche en arts et en sciences at the University of Montreal. The participants provided their written informed consent to participate in this study.

## Author Contributions

All authors listed have made a substantial, direct, and intellectual contribution to the work and approved it for publication.

## Conflict of Interest

The authors declare that the research was conducted in the absence of any commercial or financial relationships that could be construed as a potential conflict of interest.

## Publisher’s Note

All claims expressed in this article are solely those of the authors and do not necessarily represent those of their affiliated organizations, or those of the publisher, the editors and the reviewers. Any product that may be evaluated in this article, or claim that may be made by its manufacturer, is not guaranteed or endorsed by the publisher.

## Footnotes

^1^ http://beatport.com

^2^ http://songbpm.com
